# An Electrochemical Study on the Effect of Metal Chelation and Reactive Oxygen Species on a Synthetic Neuromelanin Model

**DOI:** 10.3389/fbioe.2019.00227

**Published:** 2019-10-18

**Authors:** Ri Xu, Francesca Soavi, Clara Santato

**Affiliations:** ^1^Department of Engineering Physics, Polytechnique Montréal, Montréal, QC, Canada; ^2^Dipartimento di Chimica “Giacomo Ciamician”, Alma Mater Studiorum Università di Bologna, Bologna, Italy

**Keywords:** neuromelanin (NM), antioxidant behavior, metal chelation, radical scavenging, reactive oxygen species, redox properties, hydrogen peroxide, cyclic voltammetry

## Abstract

Neuromelanin is present in the cathecolaminergic neuron cells of the *substantia nigra* and *locus coeruleus* of the midbrain of primates. Neuromelanin plays a role in Parkinson's disease (PD). Literature reports that neuromelanin features, among others, antioxidant properties by metal ion chelation and free radical scavenging. The pigment has been reported to have prooxidant properties too, in certain experimental conditions. We propose an explorative electrochemical study of the effect of the presence of metal ions and reactive oxygen species (ROS) on the cyclic voltammograms of a synthetic model of neuromelanin. Our work improves the current understanding on experimental conditions where neuromelanin plays an antioxidant or prooxidant behavior, thus possibly contributing to shed light on factors promoting the appearance of PD.

## Introduction

Melanins are a family of biopigments ubiquitous in flora and fauna. The black-brown eumelanin, red-yellow pheomelanin and neuromelanin all belong to the melanin family (Bush et al., 2006). Neuromelanin is mainly present in the cathecolaminergic neuron cells of the *substantia nigra* and *locus coeruleus* of the midbrain of primates (Marsden, 1961; Zecca et al., 2004). Neuropathological studies report on the loss of pigmented neurons of the *substantia nigra* in patients affected by Parkinson's disease (Zecca et al., 2002; Double et al., 2003, 2008). It has been proposed in the literature that neuromelanin could have a role in neurotransmission (Meredith et al., 2013). Electron microscopy studies revealed that neuromelanin has a core-shell pheomelanin-eumelanin structure (Bush et al., 2006). Consequently, eumelanin can be proposed as a chemical model of neuromelanin to study interfacial processes between neuromelanin and its surroundings. Sepia melanin is the eumelanin extracted from the ink sac of cuttlefish (Schroeder et al., 2015).

**Figure 1:**
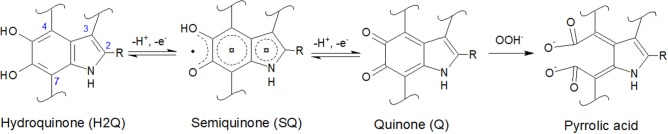
Molecular structures of 5,6-dihydroxyindole (DHI) and 5,6-dihydroxyindole-2-carboxylic acid (DHICA): R is -H in DHI and -COOH in DHICA. DHI and DHICA are building blocks of eumelanin. The redox forms of DHI and DHICA are indicated: hydroquinone (H2Q), semiquinone (SQ), and quinone (Q). OOH^−^ (the deprotonated form of H_2_O_2_, present in basic media) can oxidize quinone into pyrrolic acid (Sarna et al., 1986; Korytowski and Sarna, 1990; Smith et al., 2017).

Eumelanin is a biomacromolecule whose building blocks are 5,6-dihydroxyindole (DHI) and 5,6-dihydroxyindole-2-carboxylic acid (DHICA), co-existing in different redox states (Figure 1). Chemical synthesis permits to obtain chemically controlled DHI-melanin and DHICA-melanin, exclusively from one of the two building blocks (DHI or DHICA) (Pezzella et al., 2015). By combining well-defined amounts of the two building blocks, it is also possible to obtain DHI-DHICA-melanin, a synthetic analogous of eumelanin of interest for fundamental studies (Halliwell and Gutteridge, 1995). Eumelanin binds metal ions, such as iron and copper cations, by electrostatic interactions and/or chelation (multidentate binding). When chelated by melanin, iron and copper ions share the same binding sites, including catechol, amine and, when available, carboxylic groups (Hong et al., 2004; Di Mauro et al., 2017).

The biorole of neuromelanin is an object of debate in the scientific community (Zucca et al., 2017). On the one hand, neuromelanin could have an antioxidant behavior, e.g., by scavenging reactive oxygen species (ROS, e.g., ^•^OH and H_2_O_2_) and binding redox active transition metal ions (Liu et al., 2004; Zecca et al., 2004). Furthermore neuromelanin could feature a prooxidant behavior, e.g., by generating ROS. Neuromelanin could indeed catalyze the oxygen reduction reaction to form H_2_O_2_ (and the catalysis is reported to be more effective if melanin is engaged in iron binding) (Orive et al., 2009; Yin et al., 2014). Redox reactions possibly happen between melanin and Fe^3+^ (Fe^3+^ + H_2_Q → Fe^2+^/SQ + H^+^ or Fe^3+^ + SQ → Fe^2+^/Q + H^+^) (Pilas et al., 1988). Fe^2+^, free or chelated by melanin, can react with H_2_O_2_ to produce ^•^OH by the Fenton reaction (Fe^2+^ + H_2_O_2_ → Fe^3+^ + ^•^OH + OH^−^) (Pilas et al., 1988; Winterbourn, 1995; Zareba et al., 1995). ^•^OH can cause the degradation of neuromelanin and other molecular species in its proximity, including neurons and lipids (Pilas et al., 1988; Zecca et al., 2003; Huang et al., 2005; Brillas et al., 2009).

Electrochemical studies on neuromelanin samples in media, including transition metal ions or ROS, are expected to contribute to shed light on the nature of the experimental conditions, where neuromelanin plays an antioxidant or a prooxidant behavior. Our groups reported on the electrochemical behavior of synthetic DHI- and DHICA-melanins in the presence of NH${}_{4}^{+}$, Na^+^, K^+^, and Cu^2+^, at pH 5. We observed that DHICA-melanin voltammograms showed better resolved features than DHI-melanin in NaCH_3_COO_(aq)_, whereas DHI-melanin voltammograms featured *quasi* box-shaped behavior. In the presence of Cu^2+^, both DHICA- and DHI-melanin featured adsorption peaks (Xu et al., 2017). Zareba et al. reported that both precursors, DHI and DHICA, form melanin pigments in presence of ^•^OH (Novellino et al., 1999). Cecchi et al. reported on the quantitative comparison between free radical scavenging and redox properties of eumelanin biopigments as measured by Briggs Rausher and Folin Ciocalteu assays, to study the antioxidant activity of Sepia and synthetic melanin (Cecchi et al., 2019). Kim et al. used spectroelectrochemical reverse engineering to demonstrate that the free radical scavenging properties of Sepia and fungal melanin are affected by the redox state of the melanin (Kim et al., 2017). The same research group, with a similar approach, demonstrated that pheomelanin has higher redox-based prooxidant activity than eumelanin (Kim et al., 2015). They also reported that, with respect to bare polydopamine, the redox properties of polydopamine-Fe^3+^ complexes are strongly suppressed while those of polydopamine-Mg^2+^ complexes are maintained (Liu et al., 2019).

In this work, using the cyclic voltammetry technique, we studied the effect of metal chelation, as well as the presence of ROS moieties on the behavior of DHI-DHICA-melanin, considered as neuromelanin synthetic model, and of DHICA- and DHI-melanin. Specifically, we considered iron and copper ions (with corresponding metal-modified melanin samples named from now on as Fe/melanin, Cu/melanin, Cu/Fe/melanin) as well as H_2_O_2_ and ^•^OH (Schroeder et al., 2015). For our voltammetric studies, we used an electrolyte mimicking the intraneuronal cell solution. The morphology of the Cu/Fe/melanin samples was studied by scanning electron microscopy (SEM), whereas the chemical effect on the surface of the melanin after exposure to metals and ROS was characterized by X-ray photoelectron spectroscopy (XPS).

## Materials and Methods

### Preparation of Melanin Samples on Carbon Paper

We synthesized DHI-melanin, DHICA-melanin, DHI-DHICA-melanin (1.3:1 mol:mol) *in situ* (Pezzella et al., 1997), on carbon paper current collectors (Spectracarb™ 2050A, 10 mils) by solid-state polymerization from the corresponding building blocks (Pezzella et al., 2015). DHI and DHICA building blocks were synthesized as described hereafter (D'Ischia et al., 2005; Pezzella et al., 2015). Ten milligrams per milliliter solutions of DHI and/or DHICA monomers in methanol (99.8%, Sigma Aldrich) were prepared in ambient conditions. For DHI-DHICA-melanin, 10 mg of powder, including 5 mg of DHI monomer powder and 5 mg of DHICA monomer powder, were dissolved in methanol, in ambient conditions, and the solution was used as a precursor. The monomer solutions (5 μl) were drop cast on carbon paper featuring a geometric area of 0.5 cm^2^ (therefore the loading of the melanin samples was ca 0.1 mg cm^−2^). After drop casting, the samples were exposed overnight to NH_3_ vapors from NH_3(aq)_ (Sigma Aldrich, 28–30% w/v) to catalyze the polymerization.

### Preparation of Fe/Melanin and Cu/Fe/Melanin Samples on Carbon Paper

Samples were prepared by two different routes: route (i) and route (ii). For both routes, we prepared solutions as described in the following protocols. Fe_2_SO_4_ aqueous solutions (pH 7) were prepared from FeSO_4_·7H_2_O (≥99%, Fischer Scientific). Cu(CH_3_COO)_2_ aqueous solutions (pH 7) were prepared from Cu(CH_3_COO)_2_·H_2_O (≥98%, Sigma Aldrich). After 2 days in ambient conditions, the Fe_2_SO_4_ solutions showed yellow deposits of Fe_2_O_3_ (Beverskog and Puigdomenech, 1996). 1 M H_2_SO_4_ aqueous solutions were prepared from H_2_SO_4_ 95–98%, Sigma-Aldrich. In route (i), Fe_2_(SO_4_)_3_ aqueous solutions were adjusted at pH 3 using 1 M H_2_SO_4(aq)_ before pre-immersion (Beverskog and Puigdomenech, 1996). The effect of metal ion chelation was studied on samples obtained by route (i), based on pre-immersion of the melanin samples in solutions including copper and iron ions, and **route (ii)** based on the exposure of melanin samples to copper and iron ions present in the electrolyte where the cyclic voltammetry experiments were carried out (see Table 1). In route (i), Fe/melanin samples (0.04, 0.1, and 0.2 mol:mol) were prepared by pre-immersing melanin electrodes in 10 ml Fe_2_(SO_4_)_3_ solutions, with concentrations 1, 3, and 6 μM, for 24 h (Table 1). Cu/Fe/melanin, with molar ratio of Cu:Fe:melanin 0.002:0.2:1, were prepared by pre-immersing fresh melanin electrodes in solutions (10 ml), including 6 μM Fe_2_(SO_4_)_3_ and 0.05 μM Cu(CH_3_COO)_2_ at pH 3 (Table 1). Melanin samples obtained by the pre-immersion route were successively studied for their electrochemical properties in electrolytes free from copper and iron ions. In route (ii), we studied the effect of Fe^3+^ and Cu^2+^ following two protocols: (ii-a) 2 voltammetric cycles → addition of Fe_2_(SO_4_)_3_ with Fe:DHICA-melanin 0.04 mol:mol → 2 voltammetric cycles → addition of Fe_2_(SO_4_)_3_ with Fe:DHICA-melanin 0.14 mol:mol → 2 voltammetric cycles → addition of Fe_2_(SO_4_)_3_ with Fe:DHICA-melanin 0.23 mol:mol → 2 voltammetric cycles → addition of Cu(CH_3_COO)_2_ to form Cu/Fe/DHICA-melanin with Cu:Fe:DHICA-melanin ratio 0.002:0.23:1 mol:mol:mol; (ii-b): 2 voltammetric cycles → addition of Cu(CH_3_COO)_2_ with Cu:DHICA-melanin 0.002 mol:mol → 2 voltammetric cycles → addition of Fe_2_(SO_4_)_3_ with Cu:Fe:DHICA-melanin 0.002:0.33:1 mol:mol:mol → 2 voltammetric cycles.

**Table 1:** Concentrations of the chemical species used in this work for the corresponding experiments (volume of the solutions: 10 ml).

**Material**	**Conc. (μM)**	**Amount (nmol)**	**Material: melanin mol:mol**	**Experiment**	**Reference for the preparation of the material/solution**
Melanin	–	300	1	Melanin on carbon paper as working electrodes	Kumar et al., 2016; Xu et al., 2017
Fe^3+^	1	10	0.04	Melanin on carbon paper by pre-immersion (route i)	Zareba et al., 1995; Liu et al., 2004; Zecca et al., 2004, 2008
Fe^3+^	3	30	0.1		
Fe^3+^	6	60	0.2		
Cu^2+^	0.05	0.5	0.002		Zecca et al., 2004
Fe^3+^	1	10	0.04	Melanin on carbon paper modified by metal ions in the electrolyte (route ii)	Zareba et al., 1995; Liu et al., 2004; Zecca et al., 2004, 2008
Fe^3+^	3	40	0.14		
Fe^3+^	6	70	0.23		
Fe^3+^	10	100	0.33		
Cu^2+^	0.05	0.5	0.002		Zecca et al., 2004
Fe^3+^	48	480	0.2	Melanin on fused silica by pre-immersion (route i)	Zareba et al., 1995; Liu et al., 2004; Zecca et al., 2004, 2008
Cu^2+^	0.4	4	0.002		Zecca et al., 2004
H_2_O_2_	1,500	15,000	50	Exposure to H_2_O_2_	Chen et al., 2001
Fe^2+^	6	60	0.2	Preparation of Fenton's reagent	Zareba et al., 1995; Liu et al., 2004; Zecca et al., 2004, 2008
^•^OH	6	60	0.2	Exposure to ^•^OH	Huang et al., 2005

### Preparation of Cu/Fe/Melanin on Fused Silica

Fused silica (1 cm × 1 cm) was cleaned by sonication in acetone and water. Following a similar procedure as synthesis on carbon paper, we synthesized DHI-melanin, DHICA-melanin, DHI-DHICA-melanin (1:1 mol:mol) on fused silica by solid-state polymerization (Pezzella et al., 2015). The monomer solution (40 μl) was drop cast on 1 cm^2^ (therefore the loading of the melanin samples was ca 0.4 mg cm^−2^, four times the amount of loading on carbon paper). Cu/Fe/melanin with a molar ratio of Cu:Fe:melanin 0.002:0.2:1 were prepared on fused silica by pre-immersing fresh melanin on fused silica in solutions (10 ml) including 48 μM Fe_2_(SO_4_)_3_ and 0.4 μM Cu(CH_3_COO)_2_ at pH 3 (Table 1).

### Preparation of Solutions Containing H_2_O_2_ and ^•^OH

H_2_O_2_ (3%, for microbiology) was purchased from Sigma Aldrich. ^•^OH was prepared by the Fenton reaction (Fe^2+^ + H_2_O_2_ → Fe^3+^ + ^•^OH + OH^−^, see Table 1).

### Electrochemical Set-Up

Cyclic voltammetry was performed using a Biologic VSP 300 multichannel potentiostat, with carbon paper current collectors loaded with melanin acting as the working electrode, Pt mesh as the counter electrode and Ag/AgCl_(aq)_ (1 M KCl for Fe/melanin electrodes prepared by pre-immersion and 3 M NaCl for the rest of the work) as the reference electrode. The electrolyte (10 ml) was composed of 145 mM KCH_3_SO_4_ (99%, Acros Organics), 10 mM NaCl (≥99%, Sigma Aldrich), 2 mM MgCl_2_ (≥99%, Sigma Aldrich), 10 mM NaCH_3_COO (≥99%, Sigma Aldrich), buffered with CH_3_COOH at pH 7 (Atherton et al., 2008; Kortleven et al., 2011). The electrolyte at pH 5 was composed of 0.25 M NaCH_3_COO, buffered with CH_3_COOH.

### X-Ray Photoelectron Spectroscopy (XPS)

The XPS survey scan and high-resolution XPS analysis was carried out with a VG ESCA- LAB 3 MKII instrument under Mg Ka radiation by applying 300 W (15 kV, 20 mA) power. The pressure in the chamber during the analyses was 3.0 × 10^−9^ Torr. The high-resolution spectra were acquired with a pass energy of 20 eV and electrons were collected at a 0° takeoff angle. Peak fitting was performed with symmetrical Gaussian–Lorentzian product functions after Shirley background subtraction. Wagner sensitivity factors were used to normalize the peak intensities for quantification.

### Scanning Electron Microscopy (SEM)

SEM images were acquired at an acceleration voltage of 15 kV in the backscattered electron imaging mode using a FEI Quanta 450 Environmental Scanning Electron Microscope (FE-ESEM).

## Results and Discussion

### Cyclic Voltammograms of Melanins

**Figure 2:**
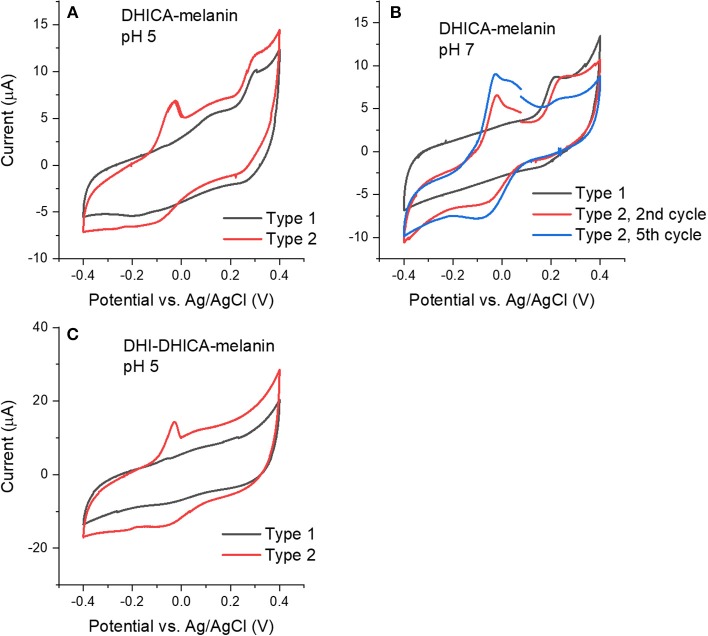
Cyclic voltammograms at 5 mV/s of **(A)** Type 1 and Type 2 DHICA-melanin in 0.25 M NaCH_3_COO pH 5, **(B)** Type 1 and Type 2 DHICA-melanin, at 5 mV/s, each for two cycles, in the simulated neurological fluid electrolyte pH 7, **(C)** Type 1 and Type 2 DHI-DHICA-melanin in 0.25 M NaCH_3_COO pH 5. DHI-mlanin only has one type of voltammogram. Only the second cycle is shown.

We initially collected cyclic voltammograms of DHICA-melanin, DHI-melanin and DHI-DHICA-melanin, in electrolytes featuring different pH, namely pH 5 and 7 (Figure 2). At pH 5, the electrolyte was 0.25 M NaCH_3_COO, selected on the basis of previous cyclic voltammetry studies carried out in our groups (Wünsche et al., 2015; Kumar et al., 2016; Xu et al., 2017). At pH 7, the electrolyte solution (described in Experimental) was selected to mimic the intraneuronal liquid (Atherton et al., 2008; Kortleven et al., 2011). Surprisingly enough, two types of cyclic voltammograms are observable, both for DHICA-melanin and DHI-DHICA-melanin (**Type 1** and **Type 2**, Figure 2), with all the experimental conditions fixed. Cyclic voltammograms of DHICA-melanin show oxidation peaks at ca 0.15 and 0.3 V vs. Ag/AgCl (3 M NaCl), at pH 5 (from now on named Type 1 DHICA-melanin, Figure 2A) and broad cathodic peaks at ca. 0.25 V and −0.2 V vs. Ag/AgCl. At pH 7, only one anodic peak is detectable at ca 0.2 V vs. Ag/AgCl for Type 1 DHICA-melanin (Figure 2B), whereas a cathodic peak is located at ca 0.2 V vs. Ag/AgCl. Type 2 DHICA-melanin shows additional anodic and cathodic peaks at both pHs. At pH 5, Type 2 DHICA-melanin features an additional sharp anodic peak at ca −0.05 V (Figure 2A) and a broad wave at ca. −0.1 V. At pH 7, Type 2 DHICA-melanin features two additional anodic peaks at ca. at ca −0.05 and 0.05 V and one additional cathodic peak at ca. −0.1 V (Figure 2B), with respect to Type 1. We tentatively explain the possibility to observe different voltammograms, for formally identical DHICA-samples, with differences in the supramolecular structure of DHICA-melanin. Such differences are attributable to the heterogeneity of the carbon paper where melanin is overgrown; carbon paper is made up of fibers and flat regions at fibers' interconnections (see later, SEM images). Results also show that cycling causes the evolution of the redox features in Type 2 DHICA-melanin, at pH 7 (Figure 2B): the intensity of the oxidation feature at ca 0.2 V decreases, whereas one of the features at ca −0.05 V increases.

**Figure 3:**
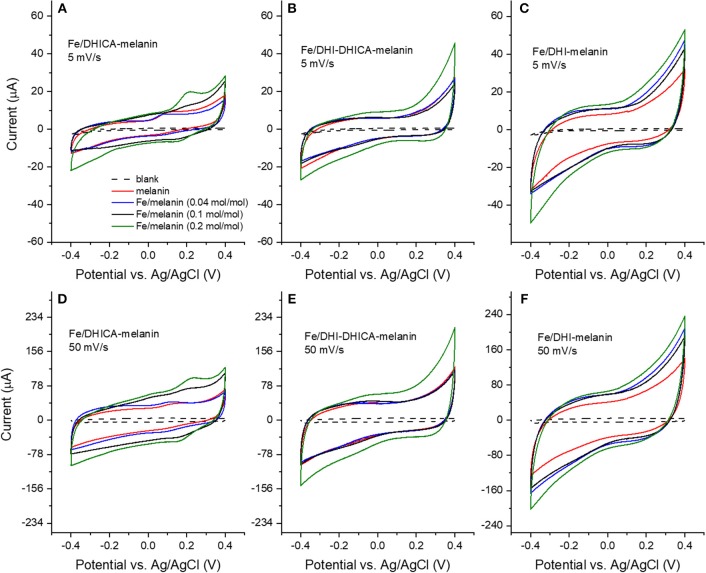
Cyclic voltammograms of Fe/melanin complexes prepared by pre-immersion at different Fe^3+^: melanin ratios: **(A,D)** Fe/DHICA-melanin (Type 1 DHICA-melanin), **(B,E)** Fe/DHI-DHICA-melanin (Type 1 DHI-DHICA-melanin) and **(C,F)** Fe/DHI-melanin. Acquisition protocol: fresh electrodes were cycled in the potential range −0.1/0.1 V, −0.2/0.2 V, −0.3/0.3 V, −0.4/0.4 V at 5 and 50 mV/s, each for two cycles in the simulated neurological fluid (pH 7) (Table 1). Only the second cycle is shown.

Voltammograms of DHI-DHICA-melanin can also feature two types of behavior, at pH 5 (from now on indicated as Type 1 and Type 2 DHI-DHICA-melanin, Figure 2C). The voltammogram of Type 1 DHI-DHICA-melanin is *quasi* box-shaped, whereas Type 2 DHI-DHICA-melanin features an oxidation peak at ca −0.05 V and a broad reduction feature. DHI-melanin features a *quasi-*box-shaped behavior, in agreement with the literature, both at pH 5 and 7 (Figures 3C,F) (Xu et al., 2017).

After the study of bare melanins, we exposed the pigment to iron and copper metal ions and ROS. The concentrations of Fe^3+^ and Cu^2+^ were chosen based on the reported concentrations of Fe:melanin ratio in the *substantia nigra* and Cu:melanin ratio in the *locus coeruleus*. The concentration of H_2_O_2_ was chosen based on the concentration reported for endogeneous H_2_O_2_ in the neuron cells (Zareba et al., 1995; Chen et al., 2001; Liu et al., 2004; Zecca et al., 2004, 2008; Huang et al., 2005).

### Cyclic Voltammograms of Fe/Melanin Prepared by Pre-immersion (Route I)

Fe^3+^ has considerably higher physiological concentrations with respect to Cu^2+^ in the brain (highest Fe:melanin ratio is ca 0.2 mol:mol in the *substantia nigra*, whereas the highest Cu:melanin ratio is 0.002 mol:mol in the *locus coeruleus*). We therefore considered, initially, Fe^3+^ for the study of the effect of metal cations on the voltammetric properties of melanin. We initially adopted the pre-immersion route (route (i), Table 1) for the preparation of the samples (Fe/DHI-melanin, Fe/DHICA-melanin and Fe/DHI-DHICA-melanin, Figure 3).

The cyclic voltammograms were obtained in solutions at pH 7 at different iron concentrations and potential sweeping rates (Figure 3). Voltammograms obtained with Fe/DHICA-melanin (Type 1 DHICA-melanin, Fe:DHICA-melanin 0.04 mol:mol) are quite similar to those of bare melanin (Figure 2 and Figures 3A,D). As the molar ratio of Fe:DHICA-melanin increases, the oxidation feature shifts anodically, to reach ca 0.2 V at 0.2 mol:mol (Figures 3A,D; Bard and Faulker, 2001). The anodic shift of the oxidation could suggest that melanin tends to have prooxidant behavior after chelating Fe^3+^ (Liu et al., 2019). We wish to remind here that an antioxidant is a substance that significantly delays or inhibits the oxidation of an oxidizable chemical substrate (Halliwell and Gutteridge, 1995). Oxidative stress is the imbalance between oxidants and antioxidants in favor of the oxidants, potentially leading to the damage of the substrate (Sies, 2000). In this context, being prooxidant means to favor the oxidative stress (Kim et al., 2015). In Fe/DHI-DHICA-melanin (Type 1 DHI-DHICA-melanin) and Fe/DHI-melanin voltammograms, no peaks are observable, unlike their bare counterparts (Figures 3B,C,E,F).

The voltammograms of DHICA-melanin, Fe/DHI-DHICA-melanin and Fe/DHI-melanin are similar at 5 and 50 mV/s (Figure 3).

We did not conduct cyclic voltammetry of Cu/melanin or Cu/Fe/melanin samples prepared by pre-immersion.

The presence of Cu^2+^ in physiological concentration (Cu:melanin 0.002 mol:mol) was not expected to affect the shape of cyclic voltammetry of melanin or Fe/melanin significantly.

### SEM Images of Cu/Fe/Melanin Prepared by Pre-immersion (Route I)

**Figure 4:**
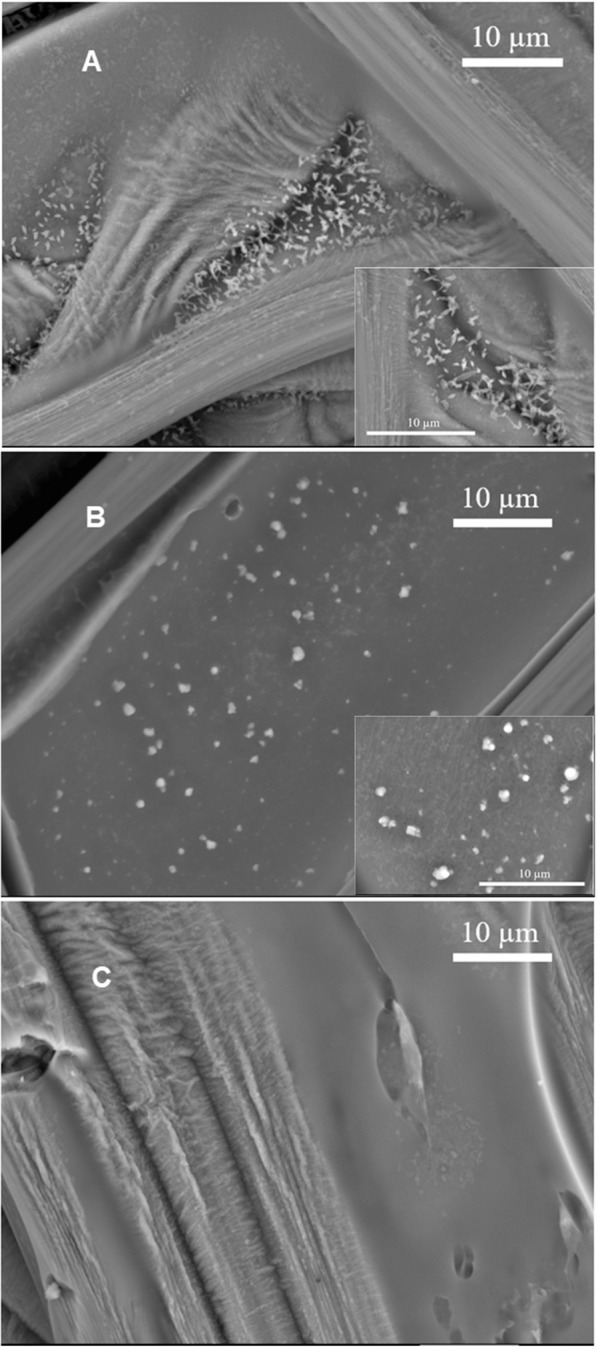
SEM images of **(A)** Cu/Fe/DHICA-melanin, **(B)** Cu/Fe/DHI-DHICA-melanin, and **(C)** Cu/Fe/DHI-melanin on carbon paper. Molar ratio of Cu:Fe:melanin 0.002:0.2:1. Backscattering mode, acceleration voltage 15 kV.

Using SEM, we investigated samples prepared by the pre-immersion route, to gain insight on the morphology of the samples. DHI-melanin, DHICA-melanin and DHI-DHICA-melanin modified with copper and iron will be indicated from now on as Cu/Fe/DHI-melanin, Cu/Fe/DHICA-melanin and Cu/Fe/DHI-DHICA-melanin. Cu/Fe/DHICA-melanin on carbon paper feature rod-shaped aggregates, mainly located at the junctions of the carbon paper fibers (Figure 4A). SEM images of Cu/Fe/DHI-DHICA-melanin show granular aggregates (Figure 4B). Rod-shaped and granular aggregates have sizes in the micrometric scale. Copper and/or iron chelation likely cause the morphological changes of DHICA- and DHI-DHICA-melanin with respect to bare melanins, where no such aggregates are observable (Figures 4A,B; Kumar et al., 2016; Xu et al., 2017). No characteristic features are observable in the SEM images of Cu/Fe/DHI-melanin (Figure 4C); this type of sample is not distinguishable from bare carbon paper, probably due to the low amount of Cu and Fe chelation in DHI-melanin (Figure 3C in Xu et al., 2017).

### XPS to Study the Presence of Metals in Cu/Fe/Melanin Prepared by Pre-immersion (Route I)

After the characterization of their morphology, samples loaded on carbon paper and fused silica, prepared by pre-immersion, were studied by XPS, to shed light on the presence of iron and copper. Iron cations are detected on Cu/Fe/melanin samples loaded on fused silica (Figure S7 and Table S2). Copper cations are barely detected here, probably due to the low concentration of Cu^2+^ used during samples' preparation [calculated to be ≤ ca. 0.018 atomic % for Cu/Fe/DHI-melanin, i.e., below the detection limit of XPS (0.1 atomic%)]. Copper and iron are barely detected on samples loaded on carbon paper (Figure S6 and Table S1), likely due to the three-dimensional, open structure of the carbon paper, which is not ideal for XPS studies. Interestingly, literature reports that iron ion-binding capacity of neuromelanin is 10-fold greater that of synthetic dopa melanin (Double et al., 2003; Costa et al., 2012).

### Effect of the Addition of Fe^3+^ to the Electrolyte on Cyclic Voltammograms of DHICA-Melanin (Route II)

Based on the more resolved voltammetric features observable with Fe/DHICA-melanin prepared by pre-immersion, with respect to Fe/DHI- and Fe/DHI-DHICA-melanins (Figure 3), we selected DHICA-melanin to study the effect of the presence of Fe^3+^ in the electrolyte solution where bare melanin samples are immersed (route ii). In this type of experiment, we added Fe_2_(SO_4_)_3_ in the electrolyte to form Fe/DHICA-melanin (Type 1 DHICA-melanin, Figures S1A, S2). Similar results as produced for pre-immersed Fe/DHICA-melanin, i.e., an anodic shift of the oxidation potential upon increase of Fe^3+^ concentration, were observed (Type 1 DHICA-melanin, Figure S1A).

### Effect of Cu^2+^ Addition in the Electrolyte to Cyclic Voltammograms of DHICA-Melanin (Route II)

**Figure 5:**
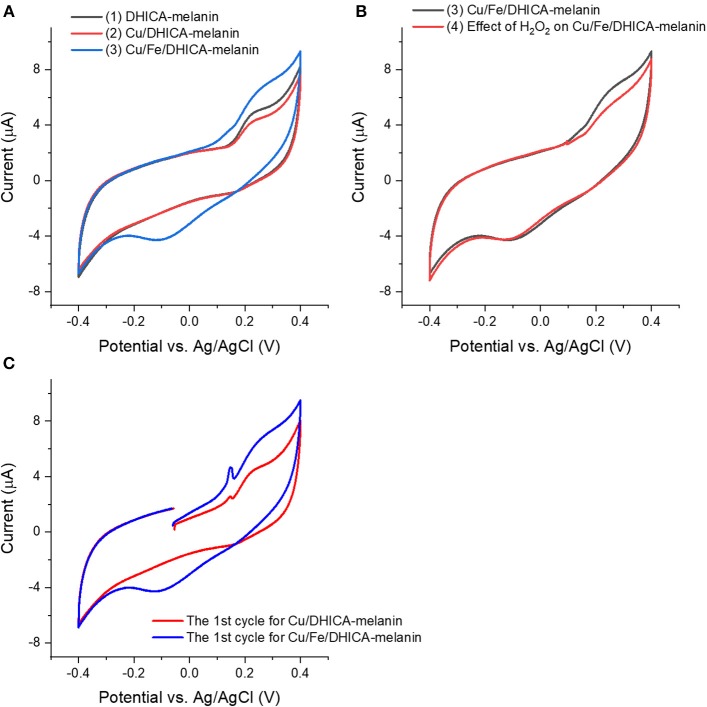
Effect of presence of **(A,C)** Cu^2+^, Fe^3+^ and **(B)** H_2_O_2_ on cyclic voltammograms of Type 1 DHICA-melanin (route ii, see Table 1), at 5 mV/s, in the simulated neurological fluid electrolyte pH 7. Protocol: 2 voltammetric cycles → add Cu(CH_3_COO)_2_ with Cu:DHICA-melanin 0.002 mol:mol → 2 voltammetric cycles → add Fe_2_(SO_4_)_3_ with Cu:Fe:DHICA-melanin 0.002:0.33:1 mol:mol:mol → 2 voltammetric cycles → expose the Cu/Fe/DHICA-melanin complex sample in H_2_O_2_ solution (0.15 mM) → 2 voltammetric cycles. Only the second cycle is shown apart from **(C)**, where the cycle reported is the first one.

We added Cu(CH_3_COO)_2_ in the electrolyte to form Cu/DHICA-melanin (route ii, Figure 5A). The presence of Cu^2+^ in physiological concentration (Cu:melanin 0.002 mol:mol) does not seem to affect the shape of the cyclic voltammogram of DHICA-melanin but an oxidation peak at ca 0.1 V in the first voltammetric cycle, attributable to the adsorption process of Cu^2+^ cations on the Type 1 DHICA-melanin (Figure 5C).

### Effect of Cu^2+^ Addition in the Electrolyte to Cyclic Voltammograms of Fe/DHICA-Melanin (Route II)

In the electrolyte, after adding Fe_2_(SO_4_)_3_ solutions to form Fe/DHICA-melanin, we added Cu(CH_3_COO)_2_ (Table 1 and Figure S1B). The presence of Cu^2+^ in physiological concentration (Cu:melanin 0.002 mol:mol) does not seem to affect the shape of the cyclic voltammogram of Fe/DHICA melanin (Figure S1B), when copper ions are added after iron ions.

### Effect of Fe^3+^ Addition in the Electrolyte to Cyclic Voltammograms of Cu/Melanin (Route II)

In the electrolyte, after adding Cu(CH_3_COO)_2_ solutions to form Cu/DHICA-melanin, we added Fe_2_(SO_4_)_3_ solution to form Cu/Fe/melanin (Figures 5A,C). Interestingly, after adding Fe^3+^ in the electrolyte, an oxidation peak at ca 0.1 V appears in the first cycle, attributable to the adsorption process of the Fe^3+^ cations on the melanin (Figure 5). The presence of high concentration of Fe^3+^ (corresponding to Fe:melanin of 0.33 mol:mol), in simultaneous presence with Cu^2+^, leads to a more pronounced oxidation between 0/0.4 V and an additional reduction wave between 0.2/−0.3 V for DHICA-melanin (Figure 5A).

### Effect of H_2_O_2_ on Melanin

**Figure 6:**
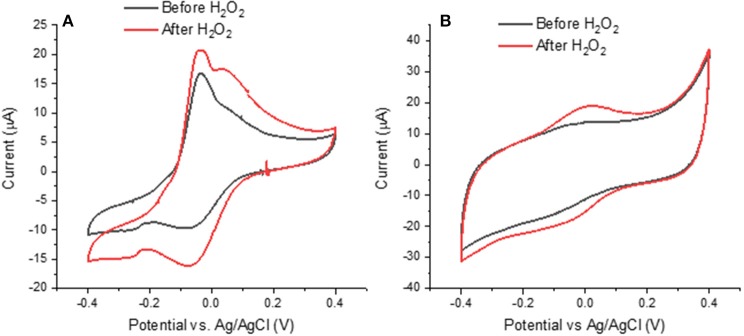
Effects of H_2_O_2_ on cyclic voltammograms of **(A)** Type 2 DHICA-melanin and **(B)** Type 1 DHI-DHICA-melanin, each for two cycles, at 5 mV/s, in a simulated neurological fluid electrolyte at pH 7 (Table 1, Fe^3+^ and Cu^2+^ are absent). Only the second cycle is shown.

Besides the effect of the presence of iron and copper ions, we studied the effect of ROS on the voltammetric behavior of bare melanin, to gain insight on a different aspect of the antioxidant behavior of melanin. Initially, we conducted experiments on the effect of exposure to H_2_O_2_ on melanin samples (Figure 6 and Figure S3).

Literature report that H_2_O_2_ can oxidize melanin into pyrrolic acids in neutral and alkaline media (Figure 1; Sarna et al., 1986; Korytowski and Sarna, 1990; Smith et al., 2017). After exposure to H_2_O_2_, Type 2 DHICA-melanin has more intense reduction and oxidation features (Figure 6A). In the case of Type 1 DHI-DHICA-melanin, H_2_O_2_ brings about the appearance of broad oxidation and reduction features at ca 0 V (Figure 6B). The cyclic voltammogram of DHI-melanin is not affected by H_2_O_2_ (Figure S3). Among other factors, the π-π stacking structure of DHI-melanin is expected to feature lower reactivity toward H_2_O_2_ with respect to DHICA-melanin, where H-bonding contributes to the formation of the supramolecular structure (Panzella et al., 2013). The effect of H_2_O_2_ on bare carbon paper is negligible (Figure S4).

### XPS to Study the Effect of H_2_O_2_ on Melanin

In order to explain the changes induced in melanin by exposure to H_2_O_2_, we used the XPS technique. For the XPS study, we considered DHI-DHICA-melanin samples (Figures S8, S9 and Table S3). Regarding changes on C 1s, ca 37% of the carbon atoms engaged in C = C disappear after exposure to H_2_O_2_ (from 26.0 to 16.3 at%). An increase of the presence of C-O (by 27%), C = O (by 43%), and O-C = O (by 17%) functional groups is observable. At the same time, the amount of C-C remains the same (Figure S9 and Table S3). These results point to the production of catechol, quinone and carboxyl group on the aromatic rings of melanin. Regarding changes involving O 1s, the presence of aliphatic C-OH groups increases by ca 23% (from 7.8 to 10.3 at%) and the presence of aromatic C-OH groups increases by ca 33% (from 5.4 to 7.2 at%). The increased amount of redox active catechol and quinone can explain the more pronounced redox features observed in DHICA-melanin and DHI-DHICA-melanin, after exposure to H_2_O_2_ (Figure 6).

### Effect of ^•^OH on Melanin

Based on the more pronounced effect of H_2_O_2_ on DHICA-melanin with respect to DHI-DHICA- and DHI-melanin, DHICA-melanin was selected to gain insight on possible effects of neuromelanin exposure to ^•^OH (Figure S5). ^•^OH was generated by the Fenton reaction (see Experimental part and Table 1). ^•^OH moieties are expected to cause the oxidation or hydroxylation of melanin (Sarna et al., 1986; Korytowski and Sarna, 1990; Huang et al., 2005; Smith et al., 2017) After exposure to ^•^OH, the behavior of Type 1 DHICA-melanin is similar to that observed after exposure to H_2_O_2_, i.e., more pronounced reduction and oxidation features (Figure S5 and Figure 6), suggesting that H_2_O_2_ and ^•^OH moieties play a similar effect on melanin. We did not observe the significant decrease of the current that we somehow expected due to melanin degradation by ^•^OH moieties at acidic and neutral pH (Pilas et al., 1988; Zareba et al., 1995; Zecca et al., 2003; Brillas et al., 2009).

### Effect of H_2_O_2_ on Cu/Fe/Melanin Prepared by Addition of the Metals to the Electrolyte (Route II)

Excessive amounts of transition metal ions bound by phenolics are reported to accelerate free-radical damage by ROS (Halliwell et al., 1995), which brought us to study the effect of H_2_O_2_ on Cu/Fe/neuromelanin. We selected Cu/Fe/DHICA-melanin (Type 1 DHICA-melanin, Figure S1B and Figure 5B) for this type of investigations. H_2_O_2_ was expected to generate ^•^OH with Cu/Fe/melanin (Pilas et al., 1988; Winterbourn, 1995; Zareba et al., 1995), due to the generation of Fe^2+^ by the redox reaction between melanin and Fe^3+^ (Fe^3+^ + H2Q or SQ → Fe^2+^/SQ or Fe^2+^/Q), in turn enabling the Fenton reaction (Pilas et al., 1988). The effect of H_2_O_2_ on the redox properties of Cu/Fe/DHICA-melanin (Type 1 DHICA-melanin) is not observable for molar ratios of 0.002:0.23:1 (Figure S1B). For higher concentrations of Fe^3+^, i.e., Fe:DHICA-melanin 0.33:1 mol:mol, the oxidation feature of Cu/Fe/DHICA-melanin (Type 1 DHICA-melanin) at ca 0.25 V tends to disappear (Figure 5B). At this concentration, H_2_O_2_ may have caused a partial degradation of Cu/Fe/melanin, in agreement with literature (Pilas et al., 1988; Zareba et al., 1995; Zecca et al., 2003; Brillas et al., 2009).

## Conclusions and Perspectives

In this work, we studied the effect of metal ions and ROS on the redox (cyclic voltammetry) properties of a neuromelanin model, in order to shed light onto possible relationships between the voltammetric properties and the antioxidant vs. prooxidant behavior of neuromelanin. Considering that literature proposes a core-shell pheomelanin-eumelanin structure for neuromelanin, we made the hypothesis that eumelanin, the shell wet by the electrolyte, is a good model to study the interfacial properties of neuromelanin and, among them, redox processes. SEM images showed the presence of changes in the morphology of DHI-DHICA-melanin and DHICA-melanin (obtained from a mix of the building blocks or exclusively from one of building block of eumelanin, DHI, and DHICA) upon the simultaneous presence of iron and copper cations, with respect to bare melanin samples. Both Cu/Fe/DHI-DHICA- and Cu/Fe/DHICA-melanin, prepared by immersing melanin electrodes in solutions of the metals, form aggregates in the micrometric range. XPS showed the presence of iron in Cu/Fe/DHI-DHICA-, Cu/Fe/DHI-, and Cu/Fe/DHICA-melanin, whereas the detection of copper was more elusive.

We observed changes in the voltammetric properties of DHICA-melanin, in the presence of iron ions: an anodic shift of the oxidation feature was observed in the voltammograms (Fe:melanin 0.2 mol:mol), which could suggest that the antioxidant properties of DHICA-melanin tend to be weaker when it chelates iron ions.

We also observed the evolution of the voltammetric properties of DHI-DHICA- and DHICA-melanins upon exposure to H_2_O_2_. The presence of H_2_O_2_ renders the voltammetric features for DHICA-melanin and DHI-DHICA-melanin more pronounced with respect to non-exposed melanin, likely due to the increase of the density of catechol and quinone groups after exposure to H_2_O_2_, in agreement with XPS results.

It has been more challenging to detect the effect of metal ions and ROS on DHI-melanin. Among other factors, the π-π stacked structure of DHI-melanin could bring lower reactivity with respect to DHICA-melanin. Indeed, literature reports that the redox properties of DHICA-melanin are attributable to the destabilizing effects of hindered intermolecular conjugation, which lead to non-planar structures with monomer-like behavior (Panzella et al., 2013).

Our results seem to suggest that DHICA is a more reactive component in eumelanin. We propose to consider the reactivity of the DHICA component, with respect to the DHI component, during studies on the loss of pigmented neurons of the *substantia nigra* in patients affected by Parkinson's disease.

In perspective, we plan to improve our neuromelanin model including a pheomelanin component in the structure and we wish to follow by time-resolved Electron Paramagnetic Resonance the effect of ROS on the charge transfer properties of neuromelanin.

## Data Availability Statement

All datasets generated for this study are included in the manuscript/Supplementary Files.

## Author Contributions

RX designed the experiments and drafted the manuscript. FS helped in the interpretation of the electrochemical measurements. CS proposed the idea, supervised the work, and helped drafting the manuscript. All the Authors gave their critical contribution on the whole manuscript.

### Conflict of Interest

The authors declare that the research was conducted in the absence of any commercial or financial relationships that could be construed as a potential conflict of interest.
