# Supplementary figures and images for: An Electrochemical Study on the Effect of Metal Chelation and Reactive Oxygen Species on a Synthetic Neuromelanin Model
Source: Front Bioeng Biotechnol. 2019 Oct 18;7:227. doi: 10.3389/fbioe.2019.00227 (PMC6813213; doi:10.3389/fbioe.2019.00227)

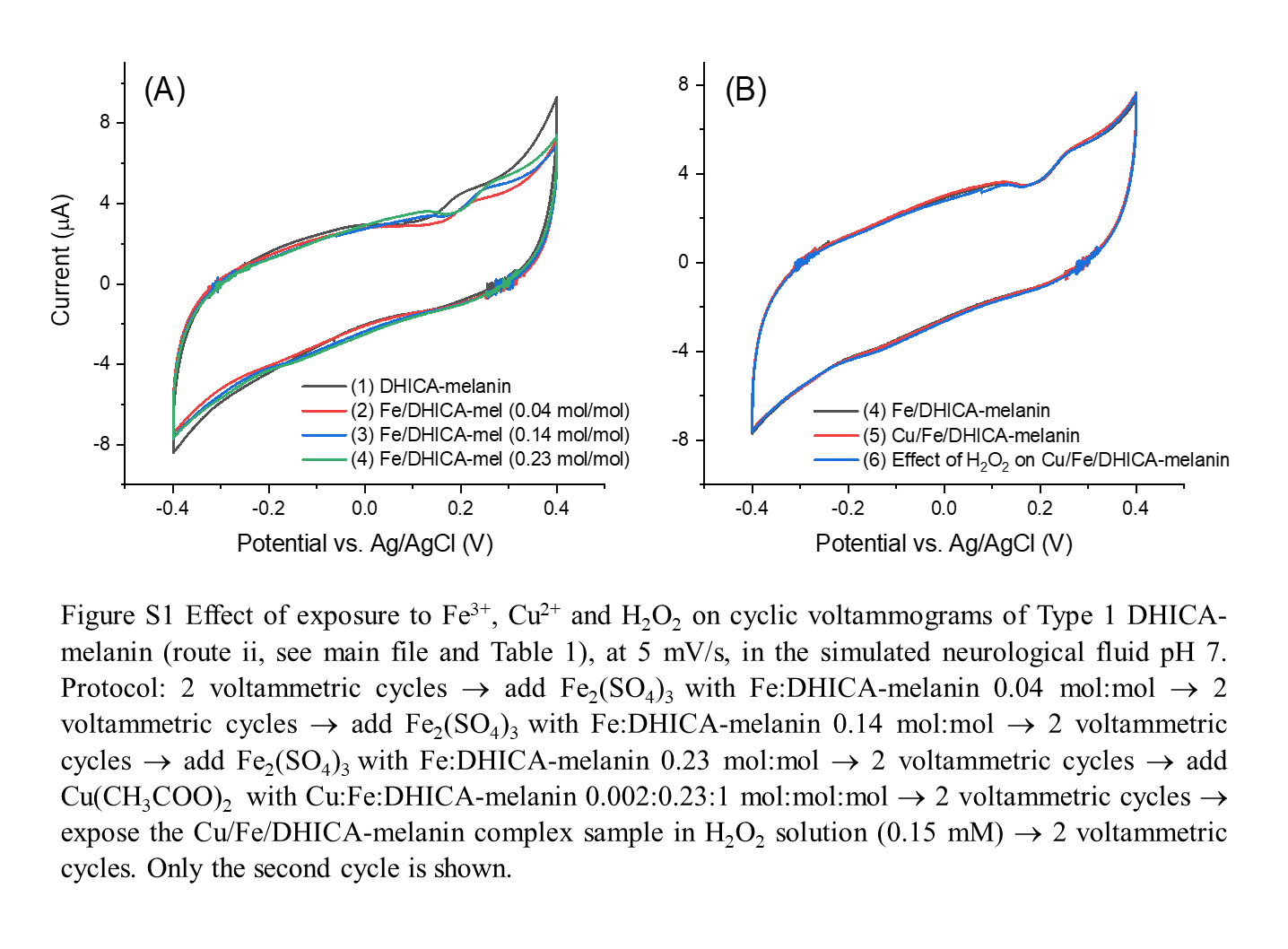

Supplement: Supplementary file 1 [file Image_1.tif]

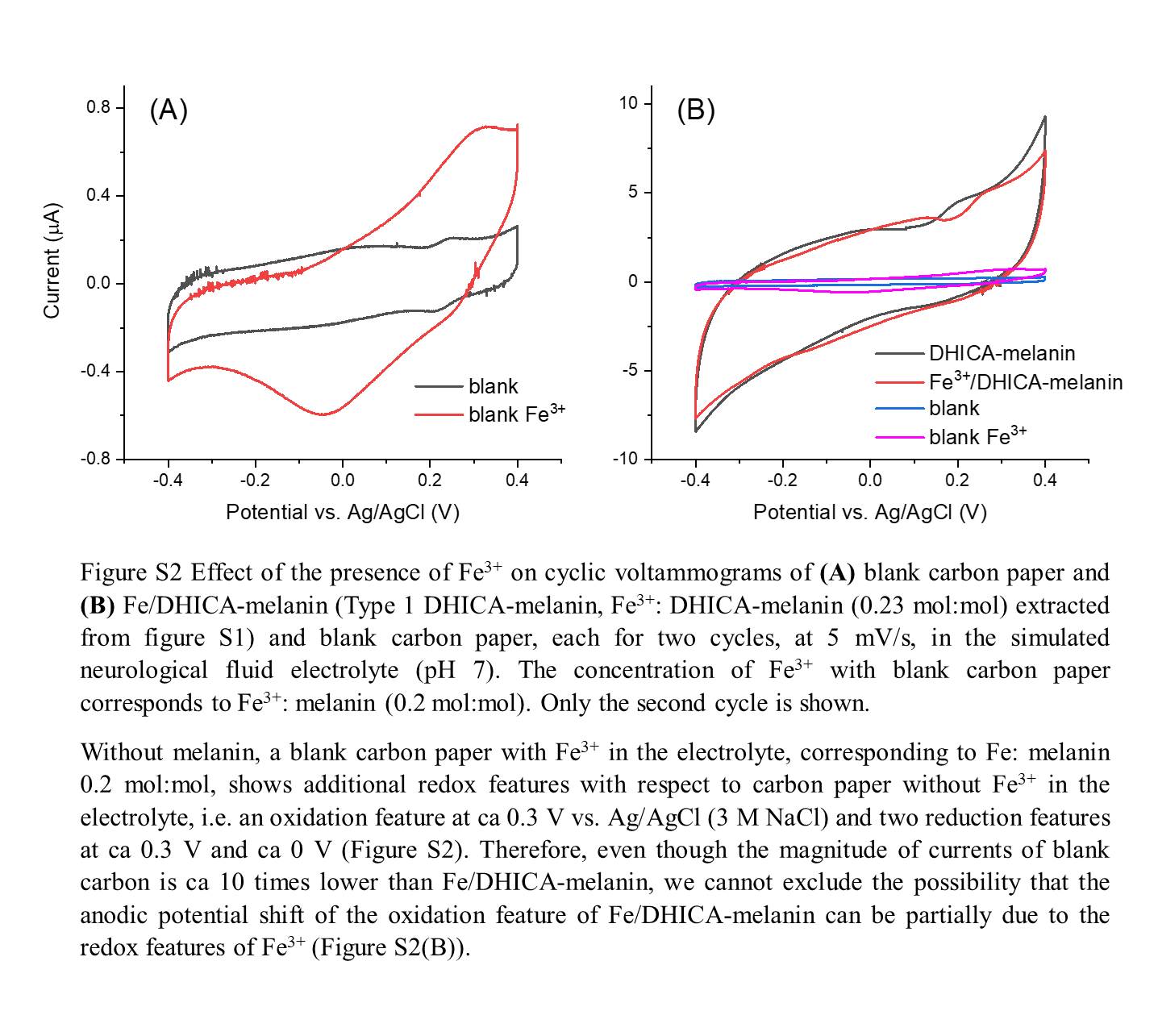

Supplement: Supplementary file 2 [file Image_2.tif]

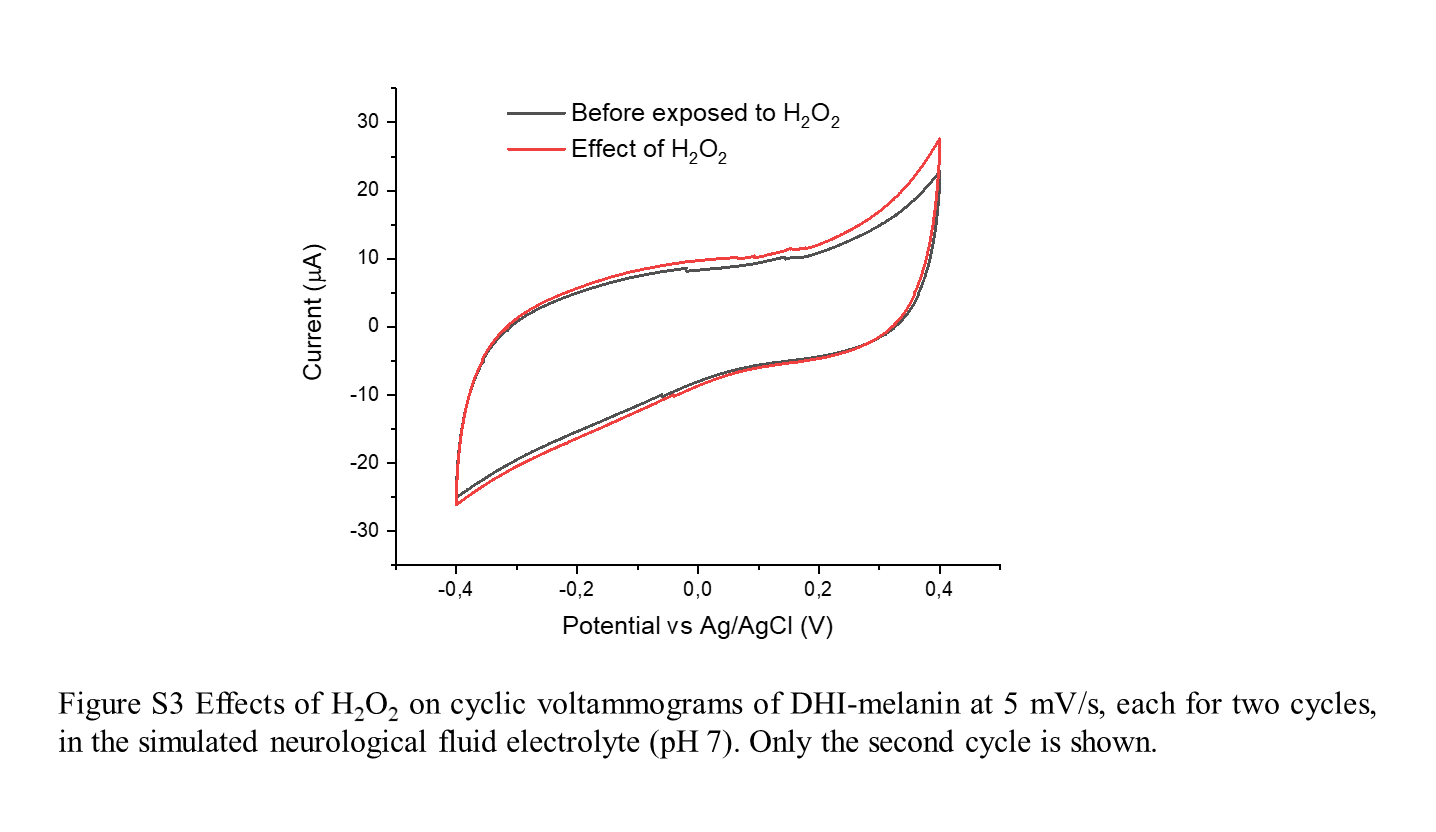

Supplement: Supplementary file 3 [file Image_3.tif]

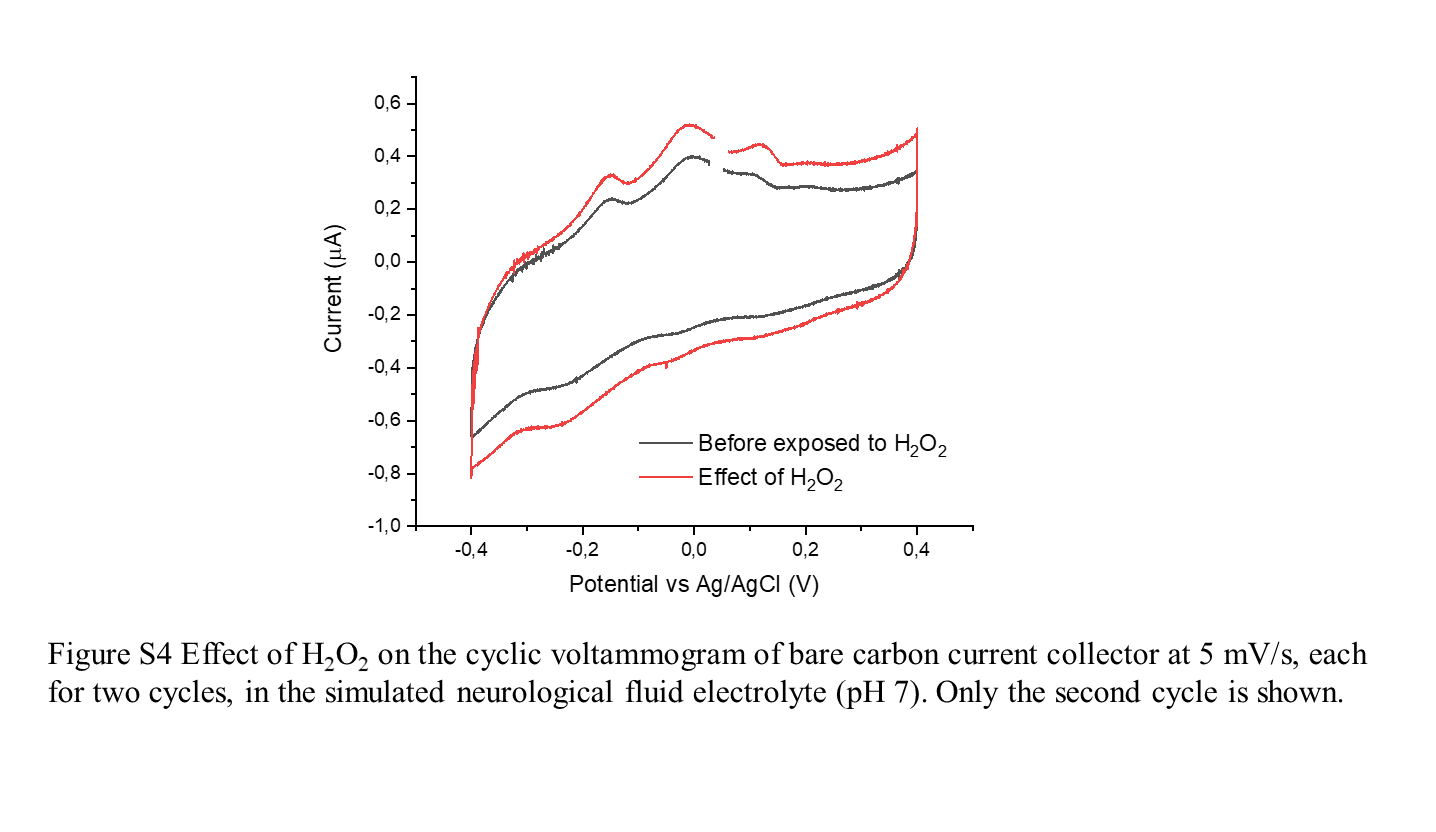

Supplement: Supplementary file 4 [file Image_4.tif]

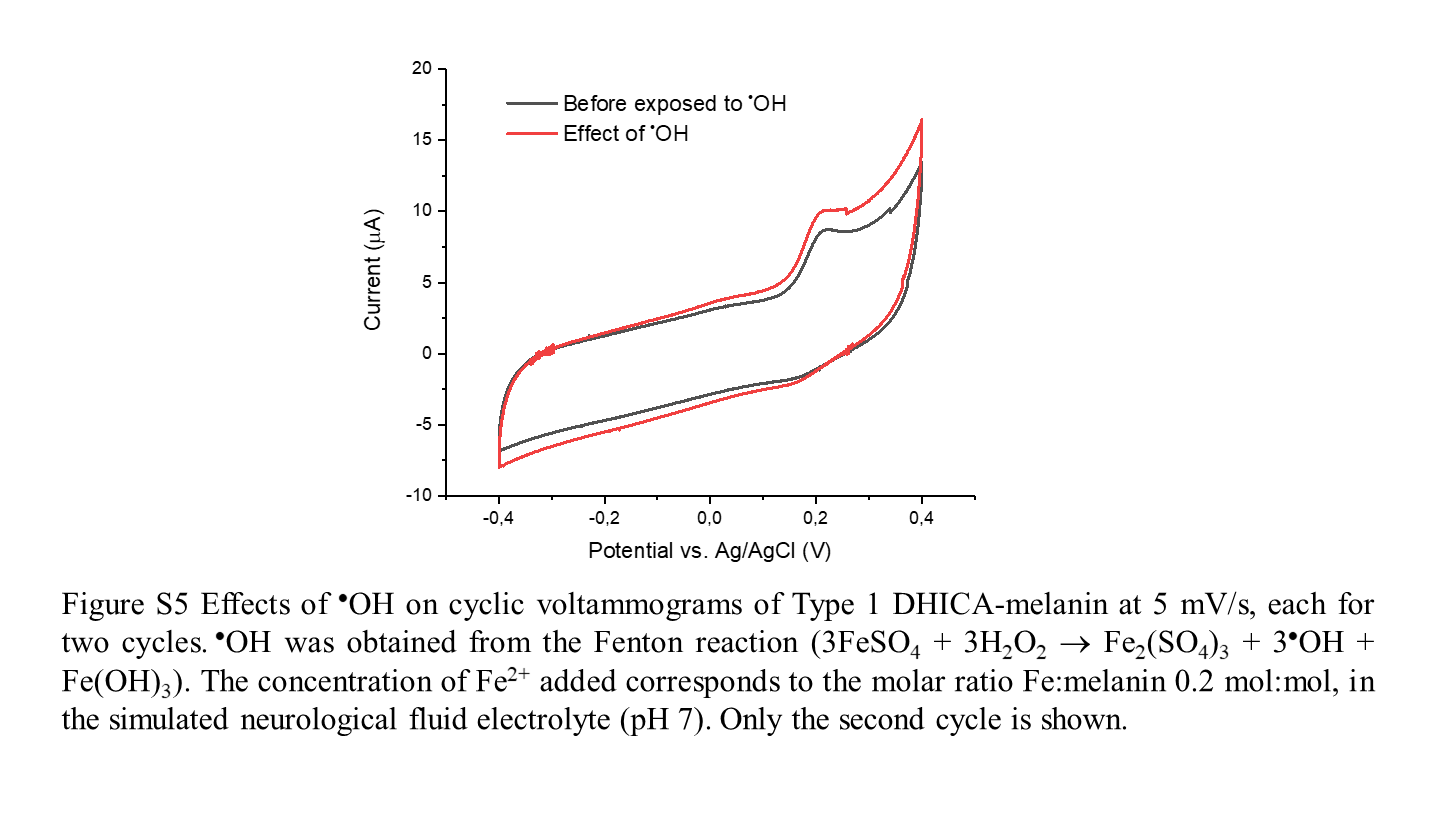

Supplement: Supplementary file 5 [file Image_5.tif]

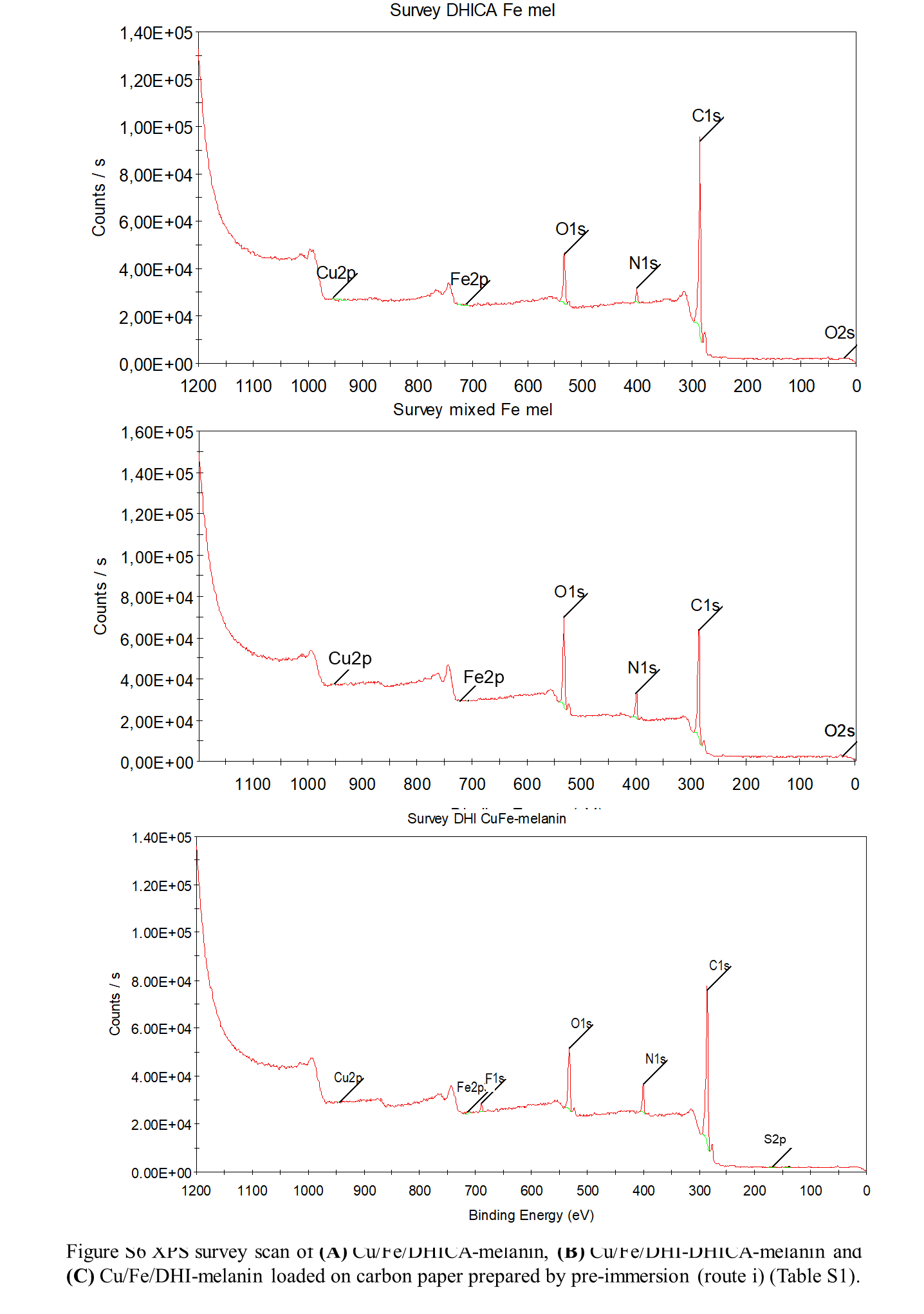

Supplement: Supplementary file 6 [file Image_6.tif]

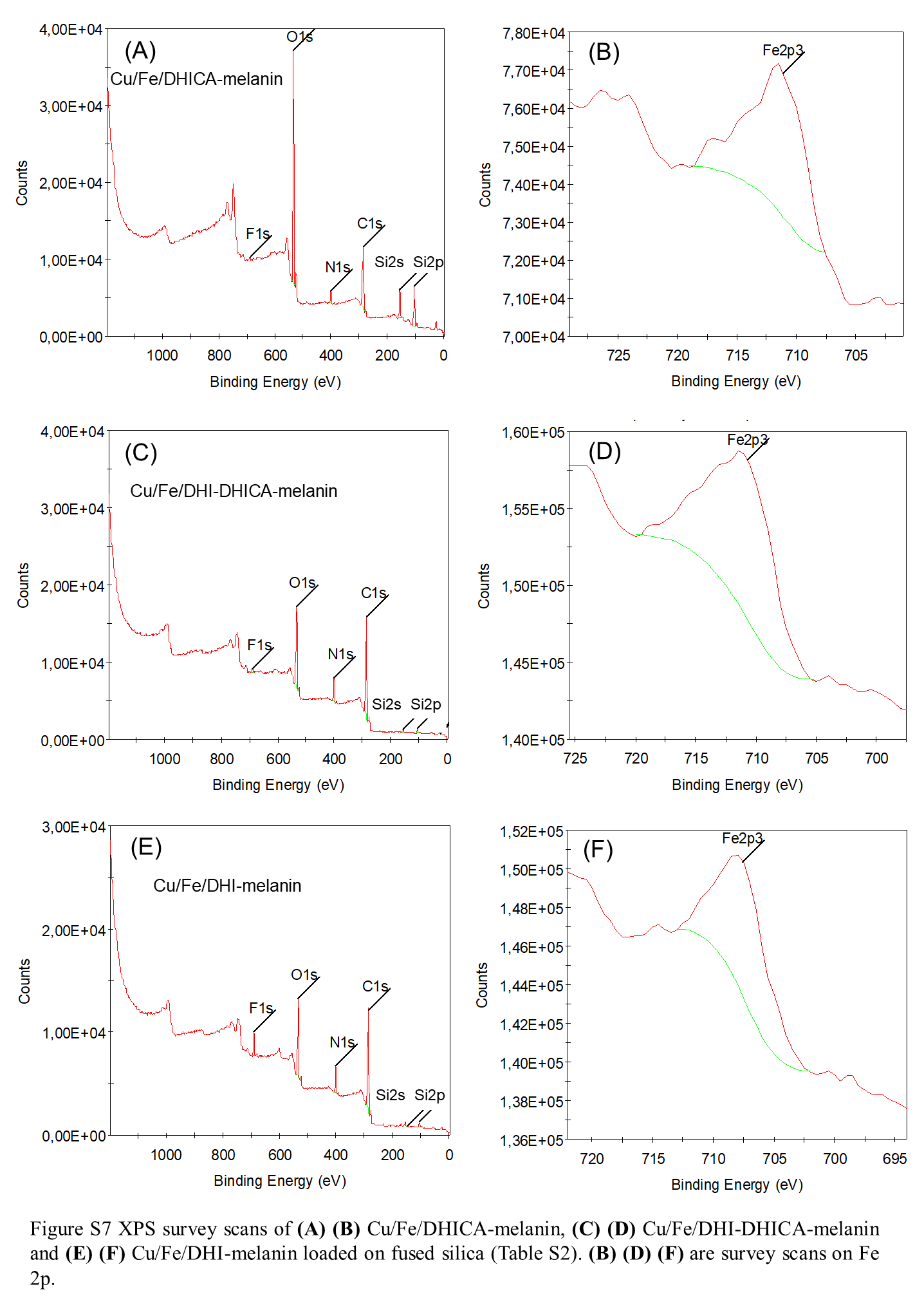

Supplement: Supplementary file 7 [file Image_7.tif]

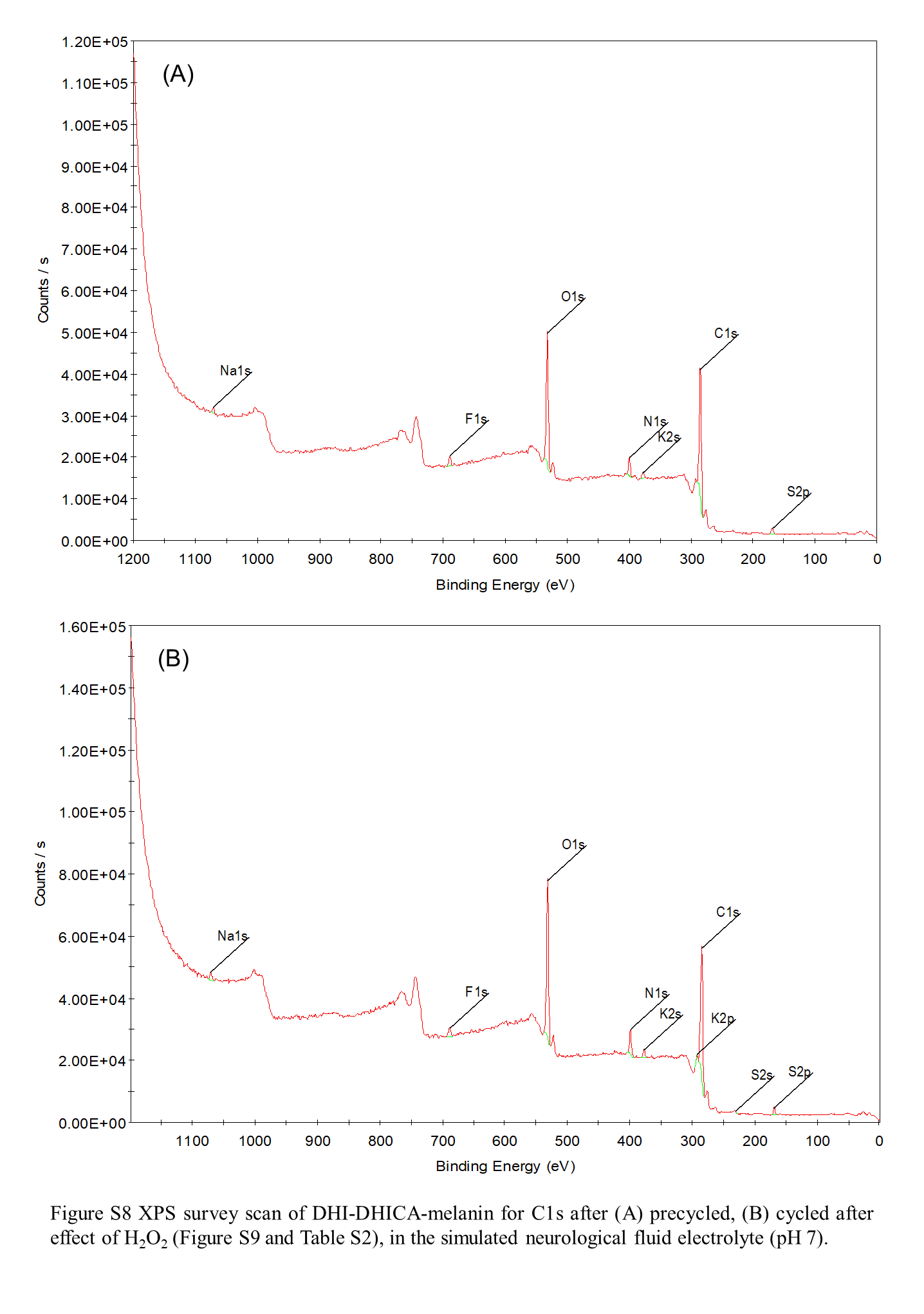

Supplement: Supplementary file 8 [file Image_8.tif]

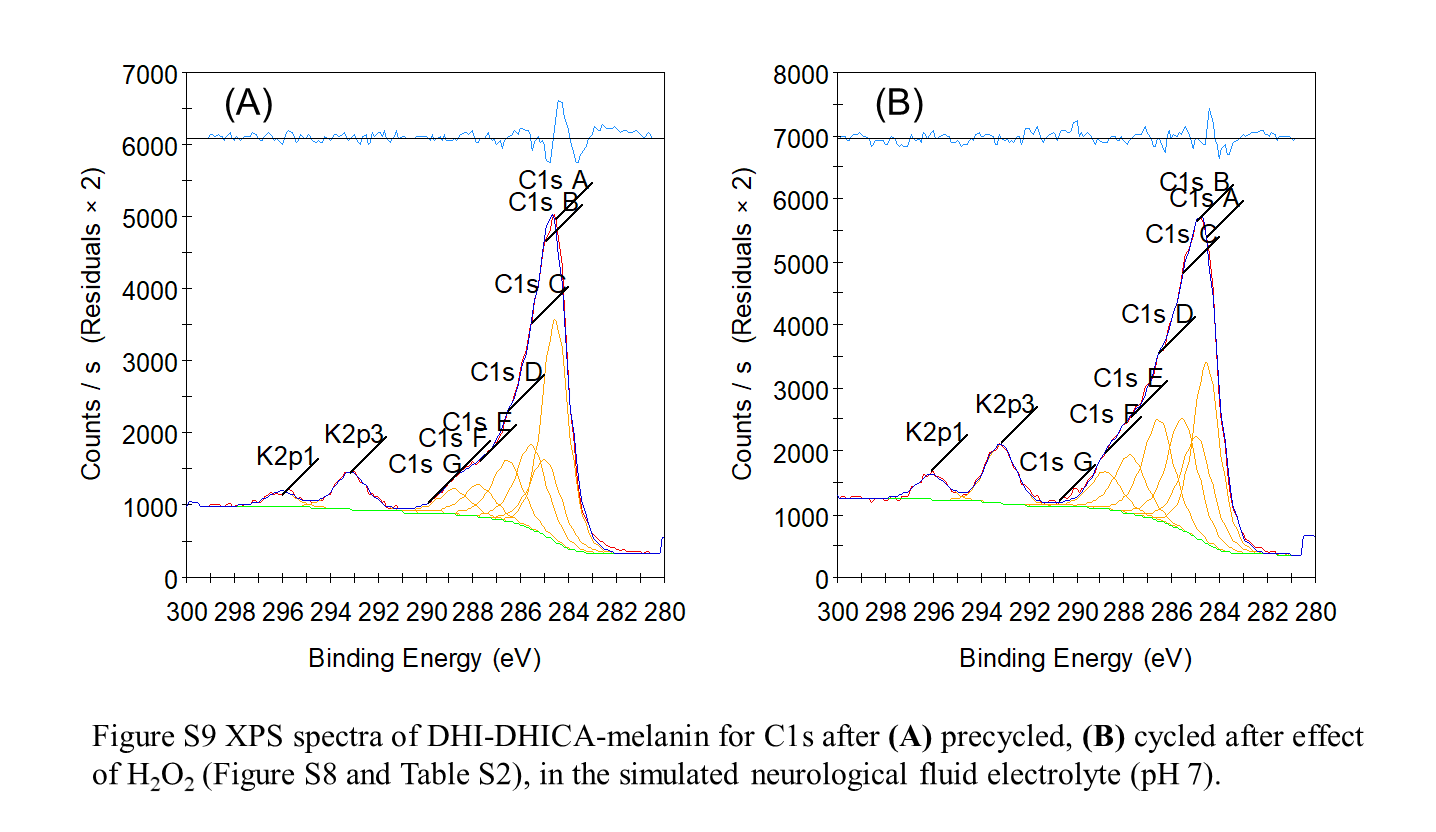

Supplement: Supplementary file 9 [file Image_9.tif]
