# Supplementary material for: An Electrochemical Study on the Effect of Metal Chelation and Reactive Oxygen Species on a Synthetic Neuromelanin Model
Source: Front Bioeng Biotechnol. 2019 Oct 18;7:227. doi: 10.3389/fbioe.2019.00227 (PMC6813213; doi:10.3389/fbioe.2019.00227)
Supplement: Supplementary file 10 [file Table_1.docx]

Table S1 Identification and quantification of elements in Cu/Fe/melanin samples loaded on carbon paper obtained by XPS survey scan (Figure S6).

| Orbital of the atom | Binding Energy (eV) | Relative Atomic % (at%) | | |
| --- | --- | --- | --- | --- |
|  |  | Cu/Fe/DHICA-melanin | Cu/Fe/DHI-DHICA-melanin | Cu/Fe/DHI-melanin |
| C 1s | 285.0 | 86.3 | 68.1 | 77.6 |
| N 1s | 399.6 | 2.9 | 8.6 | 7.4 |
| O 1s | 531.9 | 10.8 | 23.3 | 13.3 |
| Fe 2p3 | 711.2 | - | - | 0.2 |
| Cu 2p3 | 934.7 | Traces | - | - |
