# Supplementary material for: An Electrochemical Study on the Effect of Metal Chelation and Reactive Oxygen Species on a Synthetic Neuromelanin Model
Source: Front Bioeng Biotechnol. 2019 Oct 18;7:227. doi: 10.3389/fbioe.2019.00227 (PMC6813213; doi:10.3389/fbioe.2019.00227)
Supplement: Supplementary file 11 [file Table_2.docx]

Table S2 Identification and quantification of elements in Cu/Fe/melanin samples loaded on fused silica obtained by XPS survey scan (Figure S7). Results averaged over three spots.

| Orbital of the atom | Binding Energy (eV) | Relative Atomic % (at%) | | |
| --- | --- | --- | --- | --- |
|  |  | Cu/Fe/DHICA-melanin | Cu/Fe/DHI-DHICA-melanin | Cu/Fe/DHI-melanin |
| Si 2p | 104.9 | 15.4 | 2.5 | 3.4 |
| C 1s | 284.8 | 35.2 | 62.4 | 62.4 |
| N 1s | 399.6 | 3.2 | 7.0 | 8.8 |
| O 1s | 531.9 | 45.4 | 26.9 | 22.2 |
| F 1s | 688.9 | 0.3 | 1.0 | 2.9 |
| Fe 2p3 | 711.0 | 0.4 | 0.6 | 0.4 |
| Cu 2p3 | 934.7 | - | - | - |

The DHICA-melanin sample shows ca half of the amount of C 1s and N 1s with respect to DHI-melanin and DHI-DHICA-melanin, possibly due to the experimentally observed partial dissolution of the DHICA-melanin component in the pre-immersed solution (Table S2).
