# Supplementary material for: An Electrochemical Study on the Effect of Metal Chelation and Reactive Oxygen Species on a Synthetic Neuromelanin Model
Source: Front Bioeng Biotechnol. 2019 Oct 18;7:227. doi: 10.3389/fbioe.2019.00227 (PMC6813213; doi:10.3389/fbioe.2019.00227)
Supplement: Supplementary file 12 [file Table_3.docx]

Table S3 Identification of chemical bonding by high resolution XPS for DHI-DHICA-melanin (Figure S8 and S9).

| Orbital of the atom | Binding Energy (eV) | Identification | Relative Atomic % (at%) | |
| --- | --- | --- | --- | --- |
|  |  |  | DHI-DHICA-melanin  before exposure to H_2_O_2_ | DHI-DHICA-melanin  after exposure to H_2_O_2_ |
| C 1s | 284.6 | C=C | 26.0 | 16.3 |
|  | 285.0 | C-C | 10.6 | 10.9 |
|  | 285.5 | C-N | 11.7 | 12.0 |
|  | 286.5 | C-O | 8.6 | 10.9 |
|  | 287.7 | C=O | 4.4 | 6.3 |
|  | 288.8 | O-C=O | 3.5 | 4.1 |
|  | 290.7 | π→π* of C=C | 0.3 | 0.2 |
| N 1s | 398.5 | C=N | 0.4 | 0.7 |
|  | 400.2 | C-N | 5.3 | 5.1 |
| O 1s | 531.3 | C=O | 5.6 | 6.3 |
|  | 531.6 | O*=C-O | 4.8 | 4.1 |
|  | 532.3 | C-OH aliphatic | 7.8 | 10.3 |
|  | 533.0 | O=C-O* | 4.9 | 4.1 |
|  | 533.6 | C-OH aromatic | 5.4 | 7.2 |
|  | 535.5 | H_2_O | 0.7 | 1.3 |

*Indicates that the identification pertains to this atom.
